# Alcohol Consumption on the Heaviest Drinking Occasion and Hangovers during the First Dutch COVID-19 Lockdown

**DOI:** 10.3390/ijerph19074301

**Published:** 2022-04-03

**Authors:** Agnese Merlo, Noortje R Severeijns, Pauline A Hendriksen, Sarah Benson, Andrew Scholey, Johan Garssen, Gillian Bruce, Joris C Verster

**Affiliations:** 1 Division of Pharmacology, Utrecht Institute for Pharmaceutical Sciences, Utrecht University, 3584CG Utrecht, The Netherlands; a.merlo@uu.nl (A.M.); noorsevereijns@gmail.com (N.R.S.); p.a.hendriksen@students.uu.nl (P.A.H.); j.garssen@uu.nl (J.G.); 2 Centre for Human Psychopharmacology, Swinburne University, Melbourne, VIC 3122, Australia; sarahbenson@swin.edu.au; 3 Nutrition Dietetics and Food, School of Clinical Sciences, Monash University, Clayton, VIC 3168, Australia; andrew@scholeylab.com; 4 Global Centre of Excellence Immunology, Nutricia Danone Research, 3584CT Utrecht, The Netherlands; 5 Division of Psychology and Social Work, School of Education and Social Sciences, University of the West of Scotland, Paisley PA1 2BE, UK; gillian.bruce@uws.ac.uk

**Keywords:** alcohol, hangover, COVID-19, lockdown, heaviest drinking occasion, subjective intoxication, age, sex

## Abstract

The purpose of this study was to compare alcohol consumption between the heaviest drinking occasion in the period before the coronavirus disease 2019 (COVID-19) lockdown (15 January–14 March 2020) and the first COVID-19 lockdown period (15 March–11 May 2020) in the Netherlands, including the presence and severity of associated hangovers. The analysis included a sub-sample from the “Corona Lockdown: how fit are you?” (CLOFIT) study, comprising N = 761 participants who reported consuming alcohol in 2020. Overall, on the heaviest drinking occasion during the first COVID-19 lockdown period a significant reduction in number of alcoholic drinks consumed on the heaviest drinking occasion, drinking duration, and estimated BAC was observed. A significant reduction was also observed for subjective intoxication and next-day hangover severity. During the lockdown period, a significant reduction in the frequency of alcohol hangovers was reported. Several age and sex differences were observed. Specifically, men consumed significantly more alcohol than women and experienced hangovers significantly more frequently, both before and during the lockdown. With regard to age, young adults (18–35 years old) significantly reduced their alcohol intake on the heaviest drinking occasion during the lockdown and also reported lower ratings of subjective intoxication and hangover severity. No significant changes were seen for individuals above 35 years old. In conclusion, the first COVID-19 lockdown in the Netherlands was associated with reduced alcohol intake on the heaviest drinking occasion and a reduction in the severity of hangovers, particularly among young male adults.

## 1. Introduction

The coronavirus disease 2019 (COVID-19) pandemic and associated lockdowns have had a significant negative impact on mood, wellbeing, and quality of life [1,2,3]. In addition, lockdowns involved closures of bars, restaurants, and nightclubs and the cancelation of concerts and festivals, all typical places where people come together to socialize accompanied by the consumption of alcohol. As a response to lockdowns, people have adopted various behaviors to cope with the lockdown-related negative consequences, such as increased loneliness and stress. These coping strategies, amongst others, included changes in eating and sleeping patterns, daily exercise, and smoking and drinking behavior [4,5,6], and the consequences of these behavioral adaptations may either have had beneficial or negative effects on individuals’ health status and psychological wellbeing.

Studies investigating isolation in relation to infectious diseases have also shown that lockdowns have an adverse effect on psychological wellbeing and can lead to increased alcohol consumption [7,8,9]. Furthermore, studies that investigated COVID-19 lockdown effects also presented evidence of changes in weekly alcohol consumption. Specifically, it was shown that about 50% of drinkers either increased or reduced their weekly alcohol intake and the number of drinking days per week [10,11,12]. Among drinkers that increased the quantity and/or frequency of weekly alcohol consumption, there was a significant association with greater stress, reduced perceived immune fitness, and increased presence and severity of COVID-19 symptoms. These associations were not significant in drinkers that did not alter or reduce their weekly alcohol intake [10].

Research on alcohol use during the COVID-19 pandemic has focused on weekly alcohol intake but has not presented results on the heaviest drinking occasion before or during lockdown. The heaviest drinking occasion is defined as the single drinking occasion in a certain period of time on which the most alcohol is consumed. Examining the heaviest drinking occasion is important as this occasion is likely to be responsible for most negative alcohol-related consequences, such as having a hangover. In addition, in student samples it has been shown that whereas measures such as weekly alcohol consumption fluctuate in response to constantly and rapidly changing short-term academic, social, and health demands, the quantity of alcohol they consume on their weekly ‘night(s) out’ (i.e., their heaviest weekly drinking occasions) appeared to remain relatively stable [13]. For instance, students may adapt their weekly alcohol consumption prior to work submission deadlines and examinations, but not their consumption on weekly ‘nights out’. It can be questioned, however, whether these effects hold for older age groups and both sexes and whether these persist during the COVID-19 pandemic, including unprecedented changes such as lockdowns and the closure of bars and restaurants.

The alcohol hangover is the most frequently reported negative outcome of alcohol consumption [14], defined as the combination of negative mental and physical symptoms that can be experienced after a single episode of alcohol consumption, starting when the blood alcohol concentration (BAC) approaches zero [15,16]. Several studies have presented the adverse consequences linked to experiencing hangovers, including low mood, impaired cognitive performance, and impaired performance in daily activities, such as driving a car and work performance [17,18,19,20,21,22].

Various factors are associated with heavy drinking, including individual characteristics (i.e., demographics), psychological state (e.g., mood), and social environment (e.g., living situation) [23,24]. This was also found for experiencing hangovers. First of all, about 25% of drinkers report that they never experience hangovers, despite consuming large quantities of alcohol [25,26]. Second, individual differences in alcohol metabolism may impact the presence and severity of hangovers [27,28], and recently it was shown that there is no cut-off with regard to alcohol intake, but that, depending on the individual drinker, hangovers can be experienced following the consumption of even a relatively small amount of alcohol [29]. Further, sex differences have been reported in that women typically rate hangover symptoms, such as nausea and tiredness, significantly higher than men [30]. It was further found that hangover severity increases with hangover frequency [31]. However, research on hangover frequency is scarce. Studies examining age effects reported that hangover frequency declines with increasing age [32,33]. Analysis of pre-COVID-19 alcohol consumption data on the individuals that participated in the current study revealed that after correcting for the amount of alcohol consumed, both hangover frequency and hangover severity declined with increasing age [33,34]. The analysis further showed sex differences in alcohol consumption (a higher quantity and frequency in men) and found that the frequency and severity of hangovers were the greatest among younger age groups.

To date, data on alcohol hangovers during COVID-19 lockdowns have not been published. Given this gap in knowledge, the purpose of the current analysis was to compare alcohol consumption on the heaviest drinking occasion before and during the first COVID-19 lockdown in the Netherlands, including the presence and severity of associated hangovers. The data were analyzed for both sexes and different age groups were compared. Based on previous findings [30,31,32,33], it was hypothesized that these variables may have a significant impact on alcohol consumption on the heaviest drinking occasion.

## 2. Methods

Data from alcohol consumers who participated in the ‘Corona Lockdown: how fit are you?’ (CLOFIT) study were used for the current analysis [34]. The CLOFIT study comprised an online survey among the general Dutch population 18 years and older that was completed in June and July 2020, approximately 1–2 months after the first Dutch lockdown. The survey was developed in SurveyMonkey, and participants were recruited via a Facebook advertisement. The Ethics Committee of the Faculty of Social and Behavioral Sciences of Utrecht University granted ethical approval (approval code FETC17-061), and electronic informed consent was obtained from all participants. A detailed description of the survey design and methodology can be found elsewhere [34].

Demographics considered for the current analyses included age, sex, weight, and height. Body Mass Index (BMI) was computed by dividing the participant’s weight (in kilograms) by the square of the person’s height (in meters). Alcohol consumption questions were answered for the period before the lockdown (15 January–14 March 2020) and for the lockdown period (15 March–11 May 2020). With regard to the heaviest drinking occasion within each of the two periods, the number of alcoholic drinks consumed as well as the duration of drinking (h) were reported. A graphic was shown to illustrate the drinking sizes of alcoholic drinks and how to convert these into units of alcohol. The estimated blood alcohol concentration (BAC) was computed with a modified Widmark equation, taking into account sex, body weight, and total body water [35,36]. Subjective intoxication (drunkenness) was rated on an 11-point scale ranging from 0 (totally not) to 10 (extremely drunk) [37], and next-day hangover severity was assessed on a scale ranging from 0 (no hangover) to 10 (extremely severe hangover) [38]. Finally, participants reported how many hangovers they had experienced during the previous month for both periods.

Statistical analyses were conducted with SPSS (IBM Corp. Released 2013. IBM SPSS Statistics for Windows, Version 28.0. Armonk NY, USA: IBM Corp.). The mean and standard deviation (SD) were computed for demographics and all drinking outcomes. These were also computed for males and females separately. Normality was checked statistically (using the Kolmogorov–Smirnov test) and via visual inspection. Alcohol consumption data before and during lockdown were not normally distributed and therefore nonparametric statistical tests were used. Potential sex differences were tested by applying the nonparametric Independent Samples Mann–Whitney U test. Assessments of alcohol consumption on the heaviest drinking occasion before and during the COVID-19 lockdown were compared using the Wilcoxon Signed Ranks test. A Bonferroni’s correction was applied (*p* < 0.008) to account for multiple comparisons. The analyses were conducted for the overall sample as well as age separated into age groups (Group 1 (18–25 years old), Group 2 (26–35 years old), Group 3 (36–45 years old), Group 4 (46–55 years old), Group 5 (56–65 years old), Group 6 (66–75 years old), and Group 7 (>75 years old)). The Kruskal–Wallis test, including a Bonferroni’s correction for multiple comparisons, was used to compare the age groups. The interaction between age group and sex was computed using difference scores (Δ, during lockdown–before lockdown) with the Aligned Rank Transform test [39].

## 3. Results

A total of N = 761 participants reported consuming alcohol before and during the COVID-19 lockdown. The age range was 18 to 94 years old and 61.6% were women. Participant demographics are summarized in Table 1.

Alcohol consumption outcomes are summarized in Table 2. There was a significant reduction during the lockdown for all alcohol outcomes that were assessed, including the frequency and severity of reported hangovers.

### 3.1. Sex

Drinking behavior on the heaviest drinking occasion was analyzed separately for men (N = 282) and women (N = 469). The outcomes are summarized in Table 3.

Women reported that they consumed significantly less alcohol on their heaviest drinking occasion, both before and during lockdown. Women further reported significantly fewer hangovers per month for both periods. Other sex differences were not statistically significant.

### 3.2. Age

**Figure 1:**
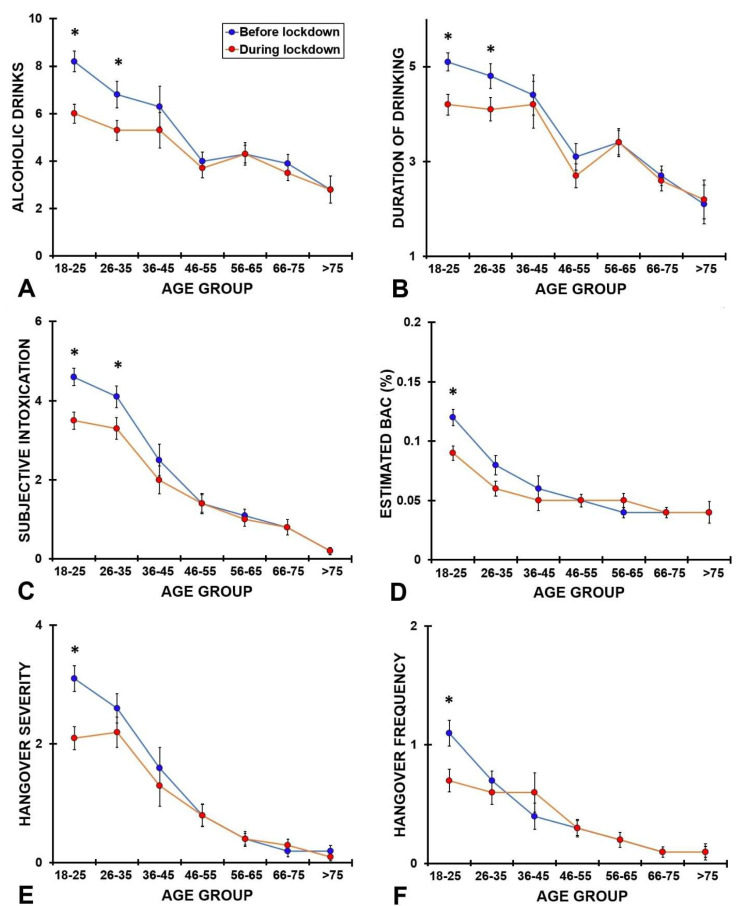
Alcohol consumption outcomes according to age group. Significant differences (*p* < 0.05) between before lockdown and during lockdown are indicated by *. Abbreviations: BAC, blood alcohol concentration.

Alcohol consumption outcomes of the different age groups are summarized in Table 4 and Table 5. The results are visualized in Figure 1. In the youngest age group (Group 1, 18–25 years old), all differences between before and during lockdown assessments were significant (*p* < 0.0001), indicating reduced alcohol intake on the heaviest drinking occasion during the COVID-19 lockdown, with lower ratings of subjective intoxication and hangover severity. This was to a lesser extent also seen among the 26–35-year-old participants (Group 2), but after Bonferroni’s correction, not all comparisons reached statistical significance. For the older age groups, no significant differences were found in drinking behavior on the heaviest drinking occasion before and during the COVID-19 lockdown.

### 3.3. Interactions between Age Group and Sex

**Figure 2:**
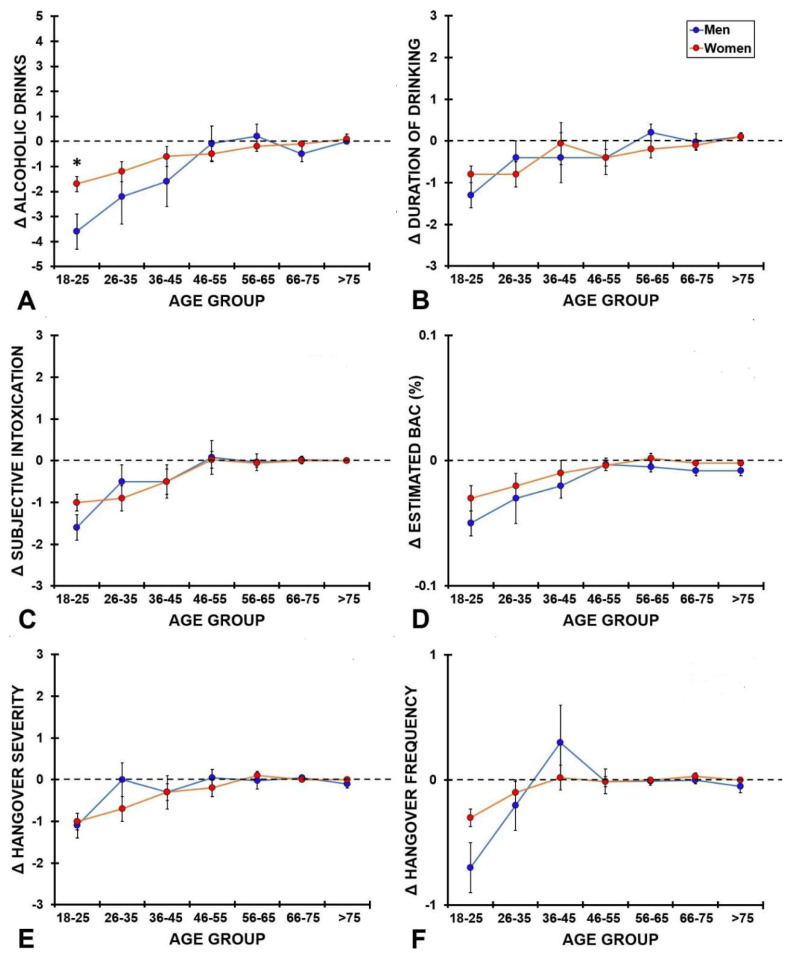
Interactions between age group and sex. Difference scores (Δ, during lockdown–before lockdown) are presented. Significant differences (*p* < 0.05) between men and women are indicated by *. Abbreviations: BAC, blood alcohol concentration.

Difference scores (Δ, during lockdown–before lockdown) were computed according to sex and age group. These are depicted in Figure 2. Significant interactions between age group and sex were found for amount of alcohol consumed (*p* < 0.001), drinking duration (*p* < 0.001), estimated BAC (*p* = 0.011), subjective intoxication (*p* < 0.001), hangover severity (*p* < 0.001), and hangover frequency (*p* < 0.001). Figure 2 shows a reduction in alcohol consumption outcomes for the younger age groups (18 to 35 years old). Thus, during the lockdown they consumed less alcohol on their heaviest drinking occasion and report lower ratings of subjective intoxication and hangover severity. In contrast, for older participants (above 35 years old) no relevant differences between before and during the COVID-19 lockdown were observed for any of the alcohol consumption outcomes. In addition, the only statistically significant sex difference was found for the number of drinks consumed (*p* < 0.001) among 18- to 25-year-old participants.

## 4. Discussion

The aim of the current study was to compare alcohol consumption on the heaviest drinking occasion before and during the first COVID-19 lockdown in the Netherlands, including the presence and severity of associated hangovers. The analysis revealed an overall reduction in alcohol consumption during the first COVID-19 lockdown. Specifically, the number of alcoholic drinks consumed on the heaviest drinking occasion, the duration of this drinking episode, subjective intoxication, and the corresponding estimated BAC were significantly reduced. A significant reduction was also observed in the frequency and severity of reported hangovers during the lockdown period. These effects were significant among younger adults, 18 to 35 years old, but not significant among older adults.

The previous literature has associated poorer mood (e.g., increased anxiety, stress, and depression) with heavy episodic drinking and with the use of alcohol as a negative reinforcement (e.g., to alleviate pain) [40,41,42]. In addition, lockdowns and their related stressors (e.g., frustration, boredom, and financial security) and individual differences in coping mechanisms, drinking motives, access to emotional support, and living situation may result in an increase in alcohol consumption [43,44,45].

As the COVID-19 lockdown is generally associated with loneliness, stress, and poor mood [43], it was expected that a general increase in heavy drinking would be observed, including the associated frequency and severity of hangovers during the COVID-19 lockdown. However, our results show the opposite effects, and significant changes in alcohol consumption outcomes between before and during the COVID-19 lockdown were only seen among 18- to 35-year-old participants. The data suggest that the closure of bars and restaurants and the canceling of events during the COVID-19 lockdown in the Netherlands had a protective effect on the episodic heavy drinking of these young adults, as the quantity of alcohol consumed, the frequency of alcohol consumption, and the frequency and severity of hangovers were significantly reduced. Recent research also suggests that our findings may be directly related to the closure of bars, restaurants, and nightclubs [46]. This Australian study [46] found a significant reduction in on-premise beer consumption when restrictions were imposed on visiting bars and restaurants, whereas no significant change in off-premise beer consumption was found during lockdown periods. Alternatively, changes in living location during the lockdown may have had an impact on the alcohol consumption of young adults. For example, White et al. reported that adolescents returning to live with their parents during a lockdown significantly reduced their daily alcohol intake compared with those that did not move back to live with their parents [44].

As expected, men consumed more alcohol than women on the heaviest drinking occasion and experienced a significantly higher frequency and severity of hangovers than women, both before and during the lockdown. These findings are in line with the general literature on alcohol consumption [47,48,49]. It is, however, important to mention that the extent of sex differences in heavy alcohol consumption varies between countries and across populations [47,48,49]. In addition, research suggests that this sex difference is rapidly declining [50,51]. Nevertheless, a statistically significant sex difference in changes in alcohol consumption outcomes during the COVID-19 lockdown was found only for the number of alcoholic drinks consumed among 18–25-year-old participants.

With regard to age, the present study showed that younger individuals (18–35 years old) significantly reduced their alcohol intake on their heaviest drinking occasion during the COVID-19 lockdown. The observation that alcohol consumption is heavier among younger age groups is in line with other studies [52,53] and is typically associated with drinking motives and contextual aspects (e.g., location, occasion, peers) that change when aging [54]. No significant changes in alcohol outcomes were seen for older participants (>35 years old). An explanation for these observations may be that younger age groups are more likely to visit bars and attend nightlife events where alcohol is consumed. Taken together, it is understandable that the observed lockdown effects on alcohol consumption were larger among younger age groups. As men consume more alcohol than women, the effects of the lockdown on alcohol consumption in young men were significantly greater than those observed among young women.

Future research should further investigate why these differences were specifically found among younger age groups, including the observed sex difference. This information could aid in the development of prevention strategies to reduce heavy drinking. Research should also continue to monitor post-COVID alcohol consumption, investigating the impact of fully reopening bars and nightlife entertainment on alcohol consumption.

The presented findings should be viewed in the context of the study’s limitations. Firstly, this study relied on self-reported retrospective data and thus responses could potentially be inaccurate. Since the assessments were made retrospectively over a period of several months, it is likely that the study outcome has been influenced to some extent by recall bias. Secondly, research relating to heavy drinking utilizes different definitions (e.g., heavy drinking, binge drinking, risky single occasion) and different measures (e.g., units or number of drinks, drink sizes). Thus, applying consistent definitions and measures is essential. In the current study, participants were provided with a description of standard serving sizes. Finally, there are several factors associated with heavy drinking and these should be taken into consideration, such as psychological states, cultural differences, and socioeconomic status. The current study aimed to identify sex and age differences associated with the first COVID-19 lockdown. However, future studies should examine the interaction with other demographics such as education level, ethnicity, health status, job type, and income.

The closure of drinking establishments (bars, restaurants, etc.) during the lockdown reduced alcohol consumption on the heaviest drinking occasion. This observation is consistent with the reduced weekly alcohol consumption of these participants that we reported elsewhere [10]. While this can be regarded as a positive effect accompanying the lockdown period, it should be noted that this is the mean outcome for the full sample under investigation. Within this sample, there is a substantial group of drinkers (about one quarter of the sample) that increased their weekly alcohol consumption during the lockdown period. While the increased consumption may have been a response to the lockdown itself, it may be that members of this subgroup increase their alcohol intake to try and mitigate the effects of negative life events more generally. This merits further investigation in contexts other than the pandemic. Further, while the reduction in alcohol consumption may be associated with positive health effects, the lockdown itself was associated with other negative health outcomes. These included significant reductions in social and psychological wellbeing, particularly among young adults [55,56]. Of note is that the observed effects on alcohol consumption are temporary and last only as long as lockdowns are enforced. Research revealed that as soon as lockdown measures were lifted, alcohol consumption patterns quickly returned to pre-COVID-19 levels [57]. 

Finally, an important implication of our findings is also relevant in a non-COVID context and concerns the general population as a whole. That is, the data show that a substantial number of participants consume too much alcohol per se. This group of drinkers deserves attention, and efforts should be made to reduce their alcohol consumption. Moderation of alcohol consumption has been associated with improved health and immune fitness ratings [10] and better academic functioning [57]. It should therefore be an important goal of policymakers to support prevention initiatives that aim to reduce heavy alcohol consumption and promote a healthy lifestyle.

## 5. Conclusions

The first COVID-19 lockdown in the Netherlands was associated with a significant reduction in the quantity of alcohol consumed on the heaviest drinking occasion. In line with this, a significant reduction in the frequency and severity of alcohol hangovers was seen during the lockdown period. These effects were most prominent in young male adults.

## Figures and Tables

**Table 1:** Demographics.

	Overall	Men	Women	*p*-Value
N (%)	761 (100%)	292 (38.4%)	469 (61.6%)	<0.001 *
Age (year)	42.3 (19.0)	48.0 (19.2)	38.7 (18.0)	<0.001 *
Weight (kg)	77.9 (16.8)	85.5 (14.9)	73.1 (16.2)	<0.001 *

Mean and standard deviation (SD, between brackets) are shown. Significant differences between men and women (*p* < 0.05) are indicated by *. Abbreviations: N, number of participants.

**Table 2:** Alcohol consumption on the heaviest drinking occasion before and during the COVID-19 lockdown.

Alcohol Consumption	Before Lockdown	During Lockdown	*p*-Value
Number of alcoholic drinks	6.0 (5.7)	4.9 (5.1)	<0.001 *
Drinking duration (h)	4.1 (2.9)	3.6 (3.0)	<0.001 *
Estimated BAC (%)	0.08 (0.09)	0.06 (0.07)	<0.001 *
Subjective intoxication	2.8 (3.1)	2.2 (2.9)	<0.001 *
Hangover severity	1.7 (2.7)	1.3 (2.4)	<0.001 *
Hangovers per month	0.6 (1.1)	0.4 (1.1)	<0.001 *

Mean and standard deviation (SD, between brackets) are shown. Significant differences between before lockdown and during lockdown (*p* < 0.008, after Bonferroni’s correction) are indicated by *. Abbreviations: BAC, blood alcohol concentration.

**Table 3:** Differences in alcohol consumption on the heaviest drinking occasion between men and women before and during the COVID-19 lockdown.

Alcohol Consumption	Before Lockdown			During Lockdown		
On the Heaviest Drinking Occasion	Men	Women	*p*-Value	Men	Women	*p*-Value
Number of alcoholic drinks	7.7 (7.1)	4.8 (4.2)	<0.0001 *	6.4 (6.2) ^†^	3.8 (3.8) ^†^	<0.001 *
Drinking duration (h)	4.4 (3.3)	3.9 (2.5)	0.180	4.1 (3.4) ^†^	3.3 (2.7) ^†^	0.009
Estimated BAC (%)	0.08 (0.09)	0.07 (0.08)	0.647	0.06 (0.07) ^†^	0.06 (0.07) ^†^	0.396
Subjective intoxication	3.0 (3.3)	2.6 (3.0)	0.182	2.5 (3.1) ^†^	2.0 (2.7) ^†^	0.048
Hangover severity	1.8 (2.7)	1.7 (2.7)	0.597	1.5 (2.6)	1.2 (2.3) ^†^	0.089
Hangovers per month	0.8 (1.4)	0.4 (0.9)	0.004 *	0.6 (1.4) ^†^	0.3 (0.8) ^†^	0.002 *

Mean and standard deviation (SD, between brackets) are shown. Significant differences between men and women (*p* < 0.008, after Bonferroni’s correction) are indicated by *. Significant differences between ‘before lockdown’ and ‘during lockdown’ (*p* < 0.008, after Bonferroni’s correction) are indicated by ^†^. Abbreviations: BAC, blood alcohol concentration.

**Table 4:** Alcohol consumption on the heaviest drinking occasion before and during the COVID-19 lockdown: a comparison among age groups.

		Number of Drinks		Drinking Duration		Estimated BAC	
Age Group (Age Range)	N	BL	DL	BL	DL	BL	DL
Group 1 (18–25)	219	8.2 (6.5)	6.0 (6.0) *	5.1 (2.8)	4.2 (3.2) *	0.12 (0.1)	0.09 (0.09) *
Group 2 (26–35)	120	6.8 (6.2)	5.3 (4.6) *	4.8 (2.9)	4.1 (2.7) *	0.08 (0.09) ^1^	0.06 (0.07)
Group 3 (36–45)	54	6.3 (6.3)	5.3 (5.5)	4.4 (3.1)	4.2 (3.6)	0.06 (0.08) ^1^	0.05 (0.06)
Group 4 (46–55)	88	4.0 (3.5) ^1,2^	3.7 (3.8) ^1,2^	3.1 (2.6) ^1,2^	2.7 (2.4) ^1,2^	0.05 (0.05) ^1^	0.05 (0.05)
Group 5 (56–65)	126	4.3 (4.2) ^1,2^	4.3 (5.3) ^1^	3.4 (2.9) ^1,2^	3.4 (3.3)	0.04 (0.05) ^1,2^	0.05 (0.07) ^1,2^
Group 6 (66–75)	85	3.9 (3.5) ^1,2^	3.5 (2.9)	2.7 (1.9) ^1,2,3^	2.6 (2.0) ^1,2^	0.04 (0.04) ^1,2^	0.04 (0.04) ^1^
Group 7 (>75)	19	2.8 (2.5) ^1,2^	2.9 (2.5)	2.1 (1.8) ^1,2^	2.2 (1.8)	0.04 (0.04) ^1^	0.04 (0.04)
Overall sample	711	6.0 (5.7)	4.9 (5.1) *	4.1 (2.9)	3.6 (3.0) *	0.08 (0.09)	0.06 (0.07) *

Mean and standard deviation (between brackets) are shown. Significant differences between ‘before lockdown’ (BL) and ‘during lockdown’ (DL) (*p* < 0.008, after Bonferroni’s correction) are indicated by *. Differences (*p* < 0.05) between age groups, after Bonferroni’s correction, are indicated as follows: ^1^, significantly different from Group 1; ^2^, significantly different from Group 2; ^3^, significantly different from Group 3.

**Table 5:** Subjective intoxication on the heaviest drinking occasion and hangover severity and frequency before and during the COVID-19 lockdown: a comparison among age groups.

		Subjective Intoxication		Hangover Severity		Hangover Frequency	
Age Group (Age Range)	N	BL	DL	BL	DL	BL	DL
Group 1 (18–25)	219	4.6 (3.2)	3.5 (3.2) *	3.1 (3.2)	2.1 (2.9) *	1.1 (1.6)	0.7 (1.4) *
Group 2 (26–35)	120	4.1 (3.0)	3.3 (3.0) *	2.6 (2.7)	2.2 (2.8)	0.7 (0.9)	0.6 (1.1)
Group 3 (36–45)	54	2.5 (2.9) ^1,2^	2.0 (2.6)	1.6 (2.5) ^1^	1.3 (2.5)	0.4 (0.8) ^1^	0.6 (1.2)
Group 4 (46–55)	88	1.4 (2.1) ^1,2^	1.4 (2.4) ^1,2^	0.8 (1.8) ^1,2^	0.8 (1.7) ^1,2^	0.3 (0.6) ^1,2^	0.3 (0.7) ^1,2^
Group 5 (56–65)	126	1.1 (1.8) ^1,2^	1.0 (1.9) ^1,2^	0.4 (1.1) ^1,2,3^	0.4 (1.4) ^1,2^	0.2 (0.7) ^1,2^	0.2 (0.7) ^1,2^
Group 6 (66–75)	85	0.8 (1.8) ^1,2,3^	0.8 (1.8) ^1,2^	0.2 (0.9) ^1,2,3^	0.3 (0.9) ^1,2^	0.1 (0.4) ^1,2^	0.1 (0.4) ^1,2^
Group 7 (>75)	19	0.2 (0.4) ^1,2^	0.2 (0.4) ^1,2^	0.2 (0.4) ^1,2^	0.1 (0.3) ^1,2^	0.1 (0.3) ^1,2^	0.1 (0.2) ^1,2^
Overall sample	711	2.8 (3.1)	2.2 (2.9) *	1.7 (2.7)	1.3 (2.4) *	0.6 (1.1)	0.4 (1.1) *

Mean and standard deviation (between brackets) are shown. Significant differences between ‘before lockdown’ (BL) and ‘during lockdown’ (DL) (*p* < 0.008, after Bonferroni’s correction) are indicated by *. Differences (*p* < 0.05) between age groups, after Bonferroni’s correction, are indicated as follows: ^1^, significantly different from Group 1; ^2^, significantly different from Group 2; ^3^, significantly different from Group 3.

## Data Availability

The survey and data are available upon request from the corresponding author.
